# *In vitro* susceptibility profile of *Plasmodium falciparum* clinical isolates from Ghana to antimalarial drugs and polymorphisms in resistance markers

**DOI:** 10.3389/fcimb.2022.1015957

**Published:** 2022-10-14

**Authors:** Wei Zhao, Xinxin Li, Qi Yang, Longcan Zhou, Mengxi Duan, Maohua Pan, Yucheng Qin, Xiaosong Li, Xun Wang, Weilin Zeng, Hui Zhao, Kemin Sun, Wenya Zhu, Yaw Afrane, Linda Eva Amoah, Benjamin Abuaku, Nancy Odurowah Duah-Quashie, Yaming Huang, Liwang Cui, Zhaoqing Yang

**Affiliations:** ^1^ Department of Pathogen Biology and Immunology, Kunming Medical University, Kunming, China; ^2^ Department of Infectious Diseases, Shanglin County People’s Hospital, Guangxi, China; ^3^ Department of Medical Microbiology, College of Health Sciences, University of Ghana, Accra, Ghana; ^4^ Noguchi Memorial Institute for Medical Research, College of Health Sciences, University of Ghana, Accra, Ghana; ^5^ Department of Protozoan Diseases, Guangxi Zhuang Autonomous Region Center for Disease Prevention and Control, Nanning, China; ^6^ Department of Internal Medicine, Morsani College of Medicine, University of South Florida, Tampa, FL, United States

**Keywords:** malaria parasite, *in vitro* assay, ring survival assay, West Africa, genetic polymorphism, drug resistance

## Abstract

Drug resistance in *Plasmodium falciparum* compromises the effectiveness of antimalarial therapy. This study aimed to evaluate the extent of drug resistance in parasites obtained from international travelers returning from Ghana to guide the management of malaria cases. Eighty-two clinical parasite isolates were obtained from patients returning from Ghana in 2016–2018, of which 29 were adapted to continuous *in vitro* culture. Their geometric mean IC_50_ values to a panel of 11 antimalarial drugs, assessed using the standard SYBR Green-I drug sensitivity assay, were 2.1, 3.8, 1.0, 2.7, 17.2, 4.6, 8.3, 8.3, 19.6, 55.1, and 11,555 nM for artemether, artesunate, dihydroartemisinin, lumefantrine, mefloquine, piperaquine, naphthoquine, pyronaridine, chloroquine, quinine, and pyrimethamine, respectively. Except for chloroquine and pyrimethamine, the IC_50_ values for other tested drugs were below the resistance threshold. The mean ring-stage survival assay value was 0.8%, with four isolates exceeding 1%. The mean piperaquine survival assay value was 2.1%, all below 10%. Mutations associated with chloroquine resistance (*pfcrt* K76T and *pfmdr1* N86Y) were scarce, consistent with the discontinuation of chloroquine a decade ago. Instead, the *pfmdr1* 86N-184F-1246D haplotype was predominant, suggesting selection by the extensive use of artemether-lumefantrine. No mutations in the *pfk13* propeller domain were detected. The *pfdhfr/pfdhps* quadruple mutant IRNGK associated with resistance to sulfadoxine-pyrimethamine reached an 82% prevalence. In addition, five isolates had *pfgch1* gene amplification but, intriguingly, increased susceptibilities to pyrimethamine. This study showed that parasites originating from Ghana were susceptible to artemisinins and the partner drugs of artemisinin-based combination therapies. Genotyping drug resistance genes identified the signature of selection by artemether-lumefantrine. Parasites showed substantial levels of resistance to the antifolate drugs. Continuous resistance surveillance is necessary to guide timely changes in drug policy.

## Introduction

According to the World Malaria Report 2021, there were 241 million malaria cases in 2020, resulting in ~627,000 deaths, a 12% increase compared with 2019 (WHO, 2021). Antimalarial therapy is one of the most important pillars of malaria control (WHO, 2015). The widespread resistance to chloroquine (CQ) and later to the antifolate drugs sulfadoxine-pyrimethamine (SP) led to the worldwide adoption of artemisinin (ART)-based combination treatments (ACTs) as the first-line treatment of uncomplicated *P. falciparum* malaria in the early 2000s (Ashley and White, 2005). However, ART resistance emerged a decade ago in the Greater Mekong Subregion of Southeast Asia (Noedl et al., 2008; Dondorp et al., 2009; Amaratunga et al., 2012; Ashley et al., 2014) and recently in East Africa (Uwimana et al., 2020; Balikagala et al., 2021; Uwimana et al., 2021; Straimer et al., 2022) is of significant concern. In Southeast Asia, the development of resistance to the ACT partner drugs mefloquine and piperaquine (PPQ) resulted in high failure rates of two first-line ACTs (Saunders et al., 2014; Leang et al., 2015; Spring et al., 2015; Amaratunga et al., 2016). With the increasing drug selection pressure due to the widespread use of ACTs, resistance monitoring is paramount to safeguard the efficacy of our last-line defense against drug-resistant *P. falciparum*.

Antimalarial drug resistance is typically monitored by determining the *in vivo* therapeutic efficacy, *in vitro*/*ex vivo* drug sensitivity, and molecular markers of resistance (Conrad and Rosenthal, 2019). *In vitro* drug assays are not influenced by host factors such as immunity and allow the detection of reduced susceptibility of *P. falciparum* to antimalarial drugs, which may be the harbinger of clinical resistance (Ataide et al., 2017). Understanding resistance mechanisms for some antimalarials provides resistance markers for molecular surveillance (Cui et al., 2015; Siddiqui et al., 2021). The *P. falciparum* CQ resistance transporter (*pfcrt*) K76T mutation is the key determinant of CQ resistance (Fidock et al., 2000; Sidhu et al., 2002). *Pfcrt* mutations also confer resistance to other 4-aminoquinolines such as amodiaquine (AQ) and PPQ (Duru et al., 2015; Agrawal et al., 2017; Ross et al., 2018; Wicht et al., 2020). *Pfmdr1* point mutations or gene amplification alter the parasite’s sensitivity to multiple drugs (Koenderink et al., 2010). The common N86Y and D1246Y mutations in Africa are linked to resistance to CQ and AQ, but increased sensitivity to lumefantrine (LMF), mefloquine, and ARTs (Duraisingh et al., 2000a; Duraisingh et al., 2000b; Reed et al., 2000). Point mutations in the dihydrofolate reductase gene (*dhfr*) (S108N, N51I, and C59R) and the dihydropteroate synthetase gene (*dhps*) (S436A, A437G, K540E, A581G, and S436F) are associated with resistance to pyrimethamine and sulfadoxine, respectively (Gregson and Plowe, 2005). In addition, the increased copy number of the *GTP cyclohydrolase 1* (*gch1*), encoding the first and rate-limiting enzyme in the folate biosynthesis pathway, has been linked to SP resistance in Southeast Asia (Nair et al., 2008). Clinical ART resistance is manifested as delayed parasite clearance (Amaratunga et al., 2012; Phyo et al., 2012; Ashley et al., 2014) and is causally linked to mutations in the propeller domain of the Kelch protein PfK13 (Ariey et al., 2014). Due to divergent antimalarial drug histories and epidemiology, drug resistance in parasite populations from different continents varies significantly. Even in the African heartland of malaria transmission, drug resistance has a high degree of heterogeneity across different geographical locations (Conrad and Rosenthal, 2019). Hard evidence documenting the intercontinental introduction and spread of drug-resistant parasites reminds us of the significance of continued surveillance for antimalarial drug resistance in sentinel sites of Africa (Wootton et al., 2002; Roper et al., 2004).

With about five million malaria cases in 2020, Ghana ranked among the top 11 highest-burden countries (WHO, 2021). In 2005, CQ use in Ghana was discontinued, and artesunate-amodiaquine (AS-AQ) was introduced as the first-line treatment, followed by the introduction of AL and dihydroartemisinin-piperaquine (DHA-PPQ) in 2008 (Abuaku et al., 2012). Even though longitudinal follow-ups of the clinical efficacy of ACTs showed that all remain highly efficacious in Ghana (Abuaku et al., 2016; Abuaku et al., 2017; Abuaku et al., 2019), molecular surveillance in Ghana showed the emergence of *pfk13* mutations, some of which have been validated to drive ART resistance *in vitro* (WHO, 2019a). In recent years, Ghana has been a major source of imported malaria cases in China, accounting for 99.7% and 58% of imported malaria cases in Shanglin County, Guangxi, in 2013 and 2016-2017, respectively (Li et al., 2015; Liu et al., 2021). Thus, resistance monitoring is critical to ensure high treatment efficacy and prevent local transmission in the recently declared malaria-free region.

In this study, we established long-term *in vitro* cultures of parasites obtained from travelers returning from Ghana. We profiled the *in vitro* susceptibilities of the parasite isolates to a panel of 11 antimalarial drugs. We also genotyped genes associated with drug resistance in order to obtain complementary information about the situation of drug resistance in the study parasite population, which will guide the local drug policy.

## Material and methods

### Ethical statement

This study was approved by the Institutional Review Board of Shanglin Hospital. Written informed consent was obtained from all volunteers.

### Parasite isolates

Patients with malaria symptoms attending the Guangxi Shanglin Hospital from 2016 to 2018, who were Chinese migrant workers that had returned from Ghana, were subjected to malaria diagnosis by microscopy using Giemsa-stained thick and thin blood smears. Patients with uncomplicated *P. falciparum* malaria were invited to donate 2-3 mL of venous blood. Those with complex travel histories to other countries besides Ghana and those who used antimalarial drugs in the previous month were excluded from the study. The blood samples in tubes with sodium citrate as the anticoagulant were transported at 4°C to the laboratory for culture adaptation.

### Culturing clinical P. falciparum isolates

The blood samples were centrifuged briefly to remove the plasma, and cell pellets were washed three times with incomplete RPMI 1640 medium buffered with 25 mM of HEPES (5.95 g/L), followed by centrifugation at 2000 rpm for 5 min to remove white blood cells. The pellet was resuspended in 10 mL of complete medium containing RPMI 1640, 2% normal human serum, 24 mM NaHCO_3_, 0.1 mM hypoxanthine, and 0.5% AlbuMAX II to produce a hematocrit of 5%, and transferred into T25 culture flasks. Parasites were cultured at 37°C in a gas mixture of 5.5% CO_2_, 2% O_2,_ and 92.5% N_2_ (Trager and Jensen, 1976; Maier and Rug, 2013). Culture media were changed every other day, and the parasite cultures were examined by microscopy daily to observe *P. falciparum* growth. On average, parasites were cultured for four-six weeks before drug assays were performed.

### *In vitro* drug assays

The antimalarial drugs used in this study included DHA, artemether (AM), AS, mefloquine, LMF, PPQ, pyronaridine, naphthoquine, pyrimethamine, and quinine. Mefloquine, pyronaridine, quinine, pyrimethamine and CQ were obtained from Sigma (St. Louis, USA); PPQ was obtained from Kangle Pharmaceutical Co., Ltd (Chongqing, China); DHA, AM, AS and naphthoquine were obtained from Kunming Pharmaceutical (Kunming, China), while LMF was obtained from Shanghai Macklin Biochemical Co., Ltd (Shanghai, China). To prepare stock drug solutions, DHA, AM, AS, LMF, mefloquine, and quinine were dissolved in absolute ethanol, and CQ, naphthoquine, and pyronaridine were dissolved in water. Pyrimethamine was dissolved in 1% acetic acid, while PPQ was prepared in 0.5% lactic acid and further diluted in water to achieve a stock solution of 320 µM. All stock solutions were stored as aliquots at -80°C. Parasites were synchronized with 5% D-sorbitol (Lambros and Vanderberg, 1979), and drug susceptibility was determined using the standard 72 h SYBR Green I-based method (Smilkstein et al., 2004). Drug stocks were first diluted in the complete medium and added to 96-well plates at the starting concentration of 100 nM for DHA, AM, and AS, 64 nM for LMF, PPQ, and naphthoquine, 640 nM for quinine, 256 nM for mefloquine, 7.5 μM for CQ, 160 nM for pyronaridine, and 750 μM for pyrimethamine, which were serially diluted. Drug assays were conducted with synchronized ring-stage parasites 2% hematocrit and 0.5% parasitemia at 37°C for 72 h. Then the plates were placed in a -80°C freezer for 30 min and thawed at room temperature. After adding 100 µL lysis solution containing 0.02% SYBR Green I (0.2 µL/mL) into each well, the plates were incubated at 37°C in the dark for 45–60 min. The plates were read with a microplate reader with excitation and emission wavelengths set at 485 and 530 nm, respectively. The half-maximal inhibitory concentration (IC_50_) of each drug was estimated using a non-linear regression model implemented in GraphPad Prism 6.

Ring survival assay (RSA) was performed using an established method (Witkowski et al., 2013; Wang et al., 2018). Briefly, tightly synchronized early ring-stage parasites (0-3 h) were treated with 700 nM of DHA or the same concentration of solvent (ethanol) for 6 h. After the drug was washed off with RPMI 1640, the parasites were cultured under standard culture conditions for 66 h. Then the surviving parasites were counted by microscopy of Giemsa-stained thin smears, with 10000 RBCs counted on each slide. The ring-stage parasite survival rates were determined by comparing surviving parasites in DHA-treated with those in vehicle-treated wells.

In addition to the standard 72 h drug assay, susceptibility of the parasites to PPQ was also measured using the PPQ survival assay (PSA) (Duru et al., 2015). Briefly, synchronized early rings (0-3 h) were adjusted to 0.5% parasitemia and exposed to 200 nM PPQ or a drug-free medium for 48 h. Then PPQ was washed off, and the parasites were cultured for an additional 24 h. Parasite survival rates were calculated by microscopically examining Giemsa-stained thin smears, with 20000 RBCs counted for each group (Witkowski et al., 2017).

The laboratory strain 3D7 was included as an internal reference for all the drug assays mentioned above. Three biological replicates and three technical replicates were performed for each parasite isolate.

### DNA extraction and sequencing of genes associated with drug resistance

DNA was extracted from the cultured parasites using QIAmp 96 DNA kit (QIAGEN, Valencia, CA, USA). Parasites were first genotyped at the *merozoite surface protein 1* (*msp1*) and *msp2* genes to determine whether the isolates were monoclonal infections (Yuan et al., 2013). Parasite DNA was used to amplify two fragments of the *pfmdr1* gene covering codons 86, 89, 184, 1226, and 1246, a *pfdhfr* fragment covering codons 51, 59, 108, and 164, a *pfcrt* fragment covering codons 72-76, and full-length *pfk13* gene (Zhao et al., 2021). Primers used are shown in **Table S1**. PCR products were purified using the EZNA Gel Extraction Kit (Omega Bio-Tek, USA) and sequenced for all strands using the Sanger sequencing method by Sangon Biotech Co. Ltd. (Kunming, China). Sequence alignments and analysis were carried out using BioEdit software 7.0. The sequences were aligned with the 3D7 sequence retrieved from PlasmoDB as the reference.

### Quantification of gch1 gene copy number

The copy number of the *pfgch1* gene was determined using a SYBR Green I-based real-time PCR method using the *pfgch1* primers (**Table S1**) and cycling conditions described previously (Osei et al., 2018). Reference samples (Dd2 and 3D7) with known *pfgch1* copy numbers and non-template negative controls were included in each run. The ΔΔCt formula (2^-ΔΔCt^) was used to estimate the relative copy numbers (Livak and Schmittgen, 2001).

### Statistical analysis

For normally distributed IC_50_ data, geometric mean and standard deviation (SD) were calculated, whereas median and interquartile range (IQR) were determined for not normally distributed data. We used t-test and Mann-Whitney U test to compare data between two groups. A *P* value of less than 0.05 was considered statistically significant. Correlations between the IC_50_s of drugs were determined using Spearman’s test in the R package.

## Results

### *In vitro* drug susceptibility

**Figure 1:**
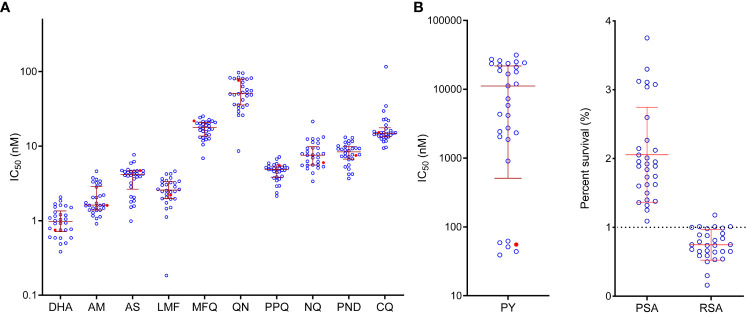
*In vitro* susceptibility of *P. falciparum* isolates to antimalarial drugs. **(A)** IC_50_ values. Each point represents the results for a single isolate. Mean IC_50_ values and SD are shown by the red horizontal bars. The red symbol represents the value of the laboratory strain 3D7. DHA, dihydroartemisinin; AM, artemether; AS, artesunate; LMF, lumefantrine; MFQ, mefloquine; QN, quinine; PPQ, piperaquine; NQ, naphthoquine; PND, pyronaridine; CQ, chloroquine; PY, pyrimethamine. **(B)** Piperaquine survival assay (PSA) and ring-stage survival assay (RSA).

We collected 82 clinical samples from malaria patients with a recent travel history to Ghana and successfully adapted 29 isolates to long-term *in vitro* culture. Genotyping at the *msp1* and *msp2* loci showed that all 29 isolates were monoclonal infections. Using the SYBR Green I assay, we profiled their *in vitro* sensitivities to 11 antimalarial drugs (**Table 1**). To make our study comparable to other *in vitro* drug susceptibility studies, we included the laboratory reference strain 3D7, the most widely used internal standard in the drug assays. In this study, the IC_50_ values of the 3D7 strain to the 11 test drugs were similar to those reported in other studies (Hao et al., 2013; Zhang et al., 2019; Wang et al., 2020). While ACTs were introduced as the first-line treatment over ten years ago, parasites were susceptible to the ART derivatives, with geometric mean IC_50_ values of 3.8, 2.1, and 1.0 nM for AS, AM, and DHA, respectively. The scatter plot also showed a relatively narrow distribution of the IC_50_ values of the field isolates to ART drugs (**Figure 1**). Compared to the reference strain 3D7, the clinical isolates only showed a significantly higher IC_50_ value for DHA (P = 0.0045). We also measured the sensitivity of the parasite isolates to DHA using RSA, with resistance defined as the RSA value exceeding 1% (Witkowski et al., 2013). While the field parasites were overall sensitive with a mean RSA value of 0.8%, four isolates had RSA values marginally higher than 1% (**Figure 1**).

**Table 1:** *In vitro* susceptibilities (IC_50_ in nM) of culture-adapted field isolates from Ghana to 11 antimalarial drugs.

Drug	3D7 (Mean ± SD)	Field isolates (n=29)		P-value^¶^
		Mean ± SD	Range	
Artemether	1.5 ± 0.2	1.6 (1.4-2.9)^a^	0.9-4.6	0.1208^b^
Artesunate	4.6 ± 0.5	3.8 ± 1.5	1.0-7.7	0.1853^c^
Dihydroartemisinin	0.6 ± 0.1	1.0 ± 0.4	0.4-2.1	0.0045^c**^
RSA(%)#	0.6 ± 0.1	0.8 ± 0.2	0.2-1.2	0.2395^c^
Lumefantrine	2.2 ± 0.3	2.7 ± 1.0	0.2-4.6	0.1923^c^
Mefloquine	22.0 ± 2.4	17.2 ± 4.4	6.9-25.2	0.0057^c**^
Piperaquine	5.8 ± 1.5	4.6 ± 1.2	2.1-7.2	0.2947^c^
PSA(%)#	1.2 ± 0.4	1.9 (1.6-2.4)^a^	1.3-3.8	0.0046^b**^
Naphthoquine	5.9 ± 0.7	7.6 (5.5-10.2)^a^	3.4-21.4	0.1532^b^
Pyronaridine	8.6 ± 1.7	8.3 ± 2.6	3.7-13.1	0.8109^c^
Chloroquine	15.2 ± 1.8	14.8 (13.3-18.1)^a^	9.4-115.9	0.8095^b^
Quinine	74.9 ± 4.5	55.1 ± 24.5	8.6-96.7	0.0313^c*^
Pyrimethamine	55.4 ± 5.0	7292 (1955-22814)^a^	39.1-31407	0.0015^b**^

^a^Data are not normally distributed and shown as median (IQR).

¶Statistical comparison between the field isolates and 3D7 was performed using Mann-Whitney U test (^b^) or t-test (^c^) * and ** indicate significance at P < 0.05 and P < 0.01, respectively.

#RSA and PSA values are percentages (%).

Five drugs tested are ACT partner drugs; AL and DHA-PPQ have been deployed in Ghana. The field isolates were relatively susceptible to these drugs, with IC_50_ values of 2.7, 4.6, 17.2, 8.3, and 8.3 nM for LMF, PPQ, mefloquine, naphthoquine, and pyronaridine, respectively (**Table 1**). The scatter plot showed that IC_50_ values for these drugs were all clustered in a relatively narrow range, with the least and most susceptible parasite isolates differing by less than seven-folds (when one outlier for LMF was excluded). For mefloquine, none of the isolates had IC_50_ values higher than the 30 nM cutoff used to define mefloquine resistance (Ringwald et al., 1996). For other drugs, the thresholds for resistance were not available. We also analyzed PPQ susceptibility using PSA, which uses 10% as the threshold for defining PPQ resistance. Our analysis indicated that all parasite isolates were sensitive to PPQ (Duru et al., 2015), with PSA ranging between 1.3 and 3.8% (**Table 1**, **Figure 1**).

Several antimalarials, including CQ, quinine, and antifolates, were previously heavily deployed in Ghana. Antifolates are still used for intermittent preventive treatment in pregnancy (IPTp) and seasonal malaria chemoprevention (SMC) in children. For CQ, 90% (26/29) of the isolates were considered susceptible, with IC_50_ values below 25 nM. Only one isolate was highly resistant (IC_50_ ≥ 100 nM), while two isolates were modestly resistant (25 nM ≤ IC_50_ < 100 nM) (**Table 1**, **Figure 1**). The geometric mean for quinine was 55.1 nM, significantly lower than that for the 3D7 strain and far below the 600 nM arbitrarily-defined threshold for resistance (Ringwald et al., 1996). For the antifolate drug pyrimethamine, two phenotypically divergent groups of isolates were identified. One group consisting of five isolates had IC_50_ values (39.1-62.2 nM) clustering near the IC_50_ value for 3D7 (55.4 nM), whereas the rest of the isolates all were highly resistant to pyrimethamine, with IC_50_ values ranging from 908 to 31,407 nM (**Figure 1**).

### Correlations between drugs

**Figure 2:**
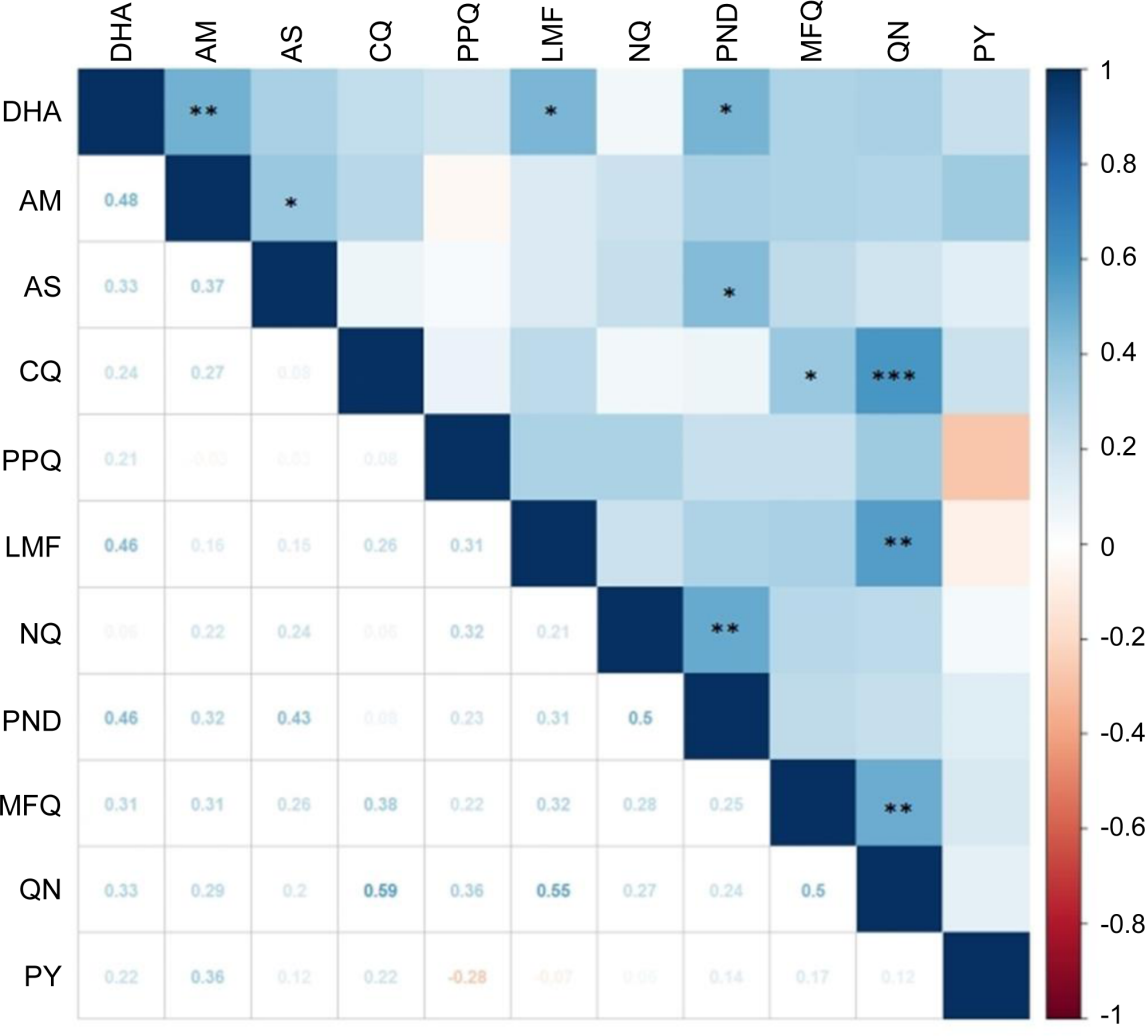
Correlation of *in vitro* susceptibility (IC_50_ values) of 29 parasite isolates to 11 antimalarial drugs analyzed by Spearman’s test. The magnitude and direction of associations between IC_50_ values are indicated by color and values. The coefficients are shown below the diagonal, while statistical significance is marked above the diagonal with *, **, and *** indicating significance at *P* < 0.05, < 0.01, and < 0.001, respectively. Drug abbreviations are the same as in **Figure 1**.

Positive correlations between *in vitro* susceptibilities to two individual drugs imply cross resistance, suggesting a similar mode of action and shared resistance mechanisms. To determine the correlations between susceptibilities to individual drugs, we made a pairwise comparison of the IC_50_ values (**Figure 2**). For the ART drugs, positive correlations were identified for AM vs. DHA (*r* = 0.48, *P* < 0.01), AM vs. AS (*r* = 0.37, *P* < 0.05), DHA vs. LMF (*r* =0.46, *P* < 0.05), DHA vs. pyronaridine (*r* =0.46, *P* < 0.05), and AS vs. pyronaridine (*r* = 0.43, *P* < 0.05). There were also positive correlations between aminoalcohol drugs: LMF vs. quinine (*r* = 0.55, *P* < 0.01), and quinine vs. mefloquine (*r* = 0.5, *P* < 0.01). In addition, CQ IC_50_s were strongly correlated with the quinine IC_50_s (*r* = 0.59, *P* < 0.001) and weakly correlated with mefloquine IC_50_s (*r* = 0.38, *P* < 0.05). The two aminoquinoline drugs, naphthoquine and pyronaridine, were also moderately correlated (*r* = 0.5, *P* < 0.01).

### Polymorphisms in drug resistance genes

We determined key mutations in *pfcrt*, *pfmdr1*, *pfk13*, *pfdhfr*, and *pfdhps* genes (**Table 2**). Only one parasite isolate had the mutant haplotype CVIET at positions 74-76, consistent with the rapid decline of the *pfcrt* mutant allele after the discontinuation of CQ. For *pfmdr1*, the N86Y and D1246Y mutations were at 6.9% and 3.4%, respectively, whereas the Y184F mutation was highly prevalent at 72.4%. The predominant 86/184/1246 haplotype is NFD (69.1%), followed by the wild type (24.1%) (**Table 3**).

**Table 2:** The prevalence of mutations (%) in genes associated with drug resistance.

Gene	Mutation	N (%)
*Pfcrt*	M74I	1 (3.4)
	N75E	1 (3.4)
	K76T	1 (3.4)
*Pfmdr1*	N86Y	2 (6.9)
	Y184F	21 (72.4)
	D1246Y	1 (3.4)
*Pfdhfr*	N51I	27 (93.1)
	C59R	28 (96.6)
	S108N	29 (100)
*Pfdhps*	I431V	1 (3.4)
	S436A/F	17 (58.6)
	A437G	26 (89.7)
	A581G	1 (3.4)
	A613S/T	3 (10.3)
*Pfk13*	K189T/N	23 (79.3)

**Table 3:** The prevalence of haplotypes of drug resistance genes.

Gene (codon positions)	Haplotypes	N (%)
*Pfcrt* (72-76)	CVMNK	28 (96.6)
	CVIET	1 (3.4)
*pfmdr1*(86/184/1246)	NYD	7 (24.1)
	NFD	20 (69.1)
	YFD	1 (3.4)
	YYY	1 (3.4)
*Pfdhfr*(51/59/108)	NRN	2 (6.9)
	IRN	26 (89.7)
	ICN	1 (3.4)
*Pfdhps*(436/437/581/613)	AGAA	11 (37.9)
	AAAA	3 (10.3)
	SGAA	12 (41.4)
	AGAS	2 (6.9)
	AGGS	1 (3.4)
*Pfdhfr/Pfdhps*	NRN-AAAA	1 (3.4)
	NRN-SGAA	1 (3.4)
	IRN-AGAA	10 (34.5)
	IRN-AAAA	2 (6.9)
	IRN-SGAA	11 (37.9)
	IRN-AGAS	2 (6.9)
	IRN-AGGS	1 (3.4)
	ICN-AGAA	1 (3.4)

Sequencing of the two antifolate resistance genes showed that the N51I, C59R, and S108N mutations in *pfdhfr* approached fixation (93-100%), resulting in a high prevalence of the triple mutation haplotype IRN (90%). The A437G mutation in *pfdhps* was also prevalent (89.7%), while S436A, A581G, and A613S/T were identified in the samples at various levels. In contrast, no 164L and 540E mutations were observed. The most predominant haplotypes SGAA and AGAA at positions 436/437/581/613 were present at 41.4 and 37.9%, respectively (**Table 3**). The most prevalent combined *dhfr/dhps* haplotypes are IRN-SGAA and IRN-AGAA, occurring at 37.9 and 34.5%, respectively. The quintuple *dhfr/dhps* mutation haplotype IRNGE at *pfdhfr* 51/59/108 and *pfdhps* 437/540 were not observed, given the lack of the 540E mutation in the study samples. Instead, the quadruple mutation haplotype IRNG (51/59/108/437) reached a 37.9% prevalence. Real-time PCR analysis of the *pfgch1* gene copy number detected five parasites (17.3%) with *pfgch1* amplification, each having three copies of *gch1* (**Table S2**). Our analysis identified that 3D7 had 3.22 copies of the *pfgch1* gene, similar to early reports (Nair et al., 2008; Heinberg et al., 2013). All parasites with *pfgch1* amplification carried the *pfdhfr* IRN triple mutations and the *pfdhps* S436A mutation, while 4/5 parasites also had the *pfdhps* A437G mutation. One also had the *pfdhps* A581G/A613S mutations.

Sequencing of the full-length *pfk13* gene did not reveal any mutations in the propeller domain. In contrast, the K189T/N mutation reached a prevalence of 79.3%.

### Association of polymorphisms with altered drug susceptibilities

We compared mutations in the genes analyzed with altered *in vitro* susceptibilities to the drugs tested. No differences were observed in IC_50_ values of all tested drugs and the *pfmdr1* 184Y and 184F alleles (*P* > 0.05) (**Table S3**). In addition, the K189T/N mutations were not associated with IC_50_ changes to the three ART derivatives or changes in the RSA value (**Table S4**).

**Figure 3:**
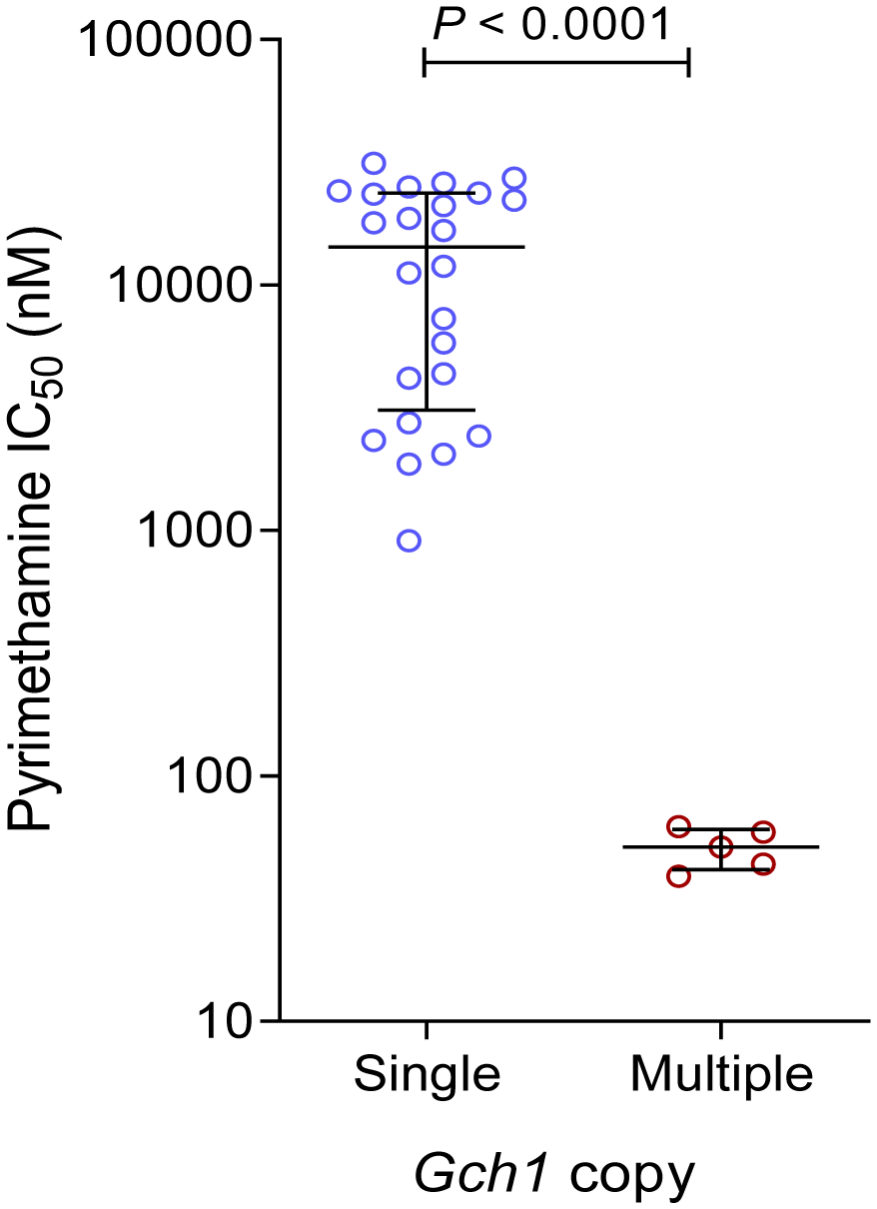
Comparison of *in vitro* susceptibilities (IC_50_ values) to pyrimethamine between parasites with a single copy and multiple copies of *pfgch1* gene.

We also compared the *gch1* gene copy number with susceptibility to pyrimethamine. Surprisingly, all five isolates with multicopy *gch1* were sensitive to pyrimethamine with IC_50_s of 39.1-62.2 nM, which were not different from that for 3D7 (55.4 nM), but significantly lower than the mean pyrimethamine IC_50_ value (13,951 nM) for isolates with a single *pfgch1* copy (**Figure 3**).

## Discussion

*In vitro* evaluation of susceptibility of malaria parasites to antimalarial drugs and molecular surveillance of drug resistance genes are complementary measures of *in vivo* therapeutic efficacy studies. Not affected by host factors, these methods may help identify earlier signs of resistance development before clinical resistance emerges. Here we established 29 long-term cultures of clinical isolates of *P. falciparum* originating from Ghana and comprehensively assessed their *in vitro* susceptibilities to a panel of 11 antimalarial drugs and genotyped five genes associated with drug resistance. Our data indicate that the parasites from Ghana are susceptible to ART drugs and most ACT partner drugs but highly resistant to antifolate drugs.

WHO reported high efficacy rates (>95%) of AL, AS-AQ, and DHA-PPQ for *P. falciparum* between 2010-2018 (WHO, 2019b). However, partial ART resistance recently detected in Uganda and Rwanda is a global public health emergency (Uwimana et al., 2020; Asua et al., 2021; Balikagala et al., 2021). Ghana adopted ACTs as the frontline treatment of uncomplicated falciparum malaria in 2005. Subsequent efficacy studies conducted in many sentinel sites of Ghana showed high efficacies of AL and AS-AQ with 28-day PCR-corrected cure rates above 90% (Abuaku et al., 2012; Abuaku et al., 2016; Abuaku et al., 2017; Abuaku et al., 2019; Abuaku et al., 2021). In addition, the imported falciparum cases among the Chinese workers returning from Ghana also responded well to the DHA-PPQ treatment with a 100% 28-day cure rate (not shown). Consistently, the *in vitro* assay showed high susceptibility of the parasite isolates to all ART family drugs, with IC_50_ values clustering in a relatively narrow range. This is drastically different from the parasites collected from Southeast Asia with wide ranges of IC_50_s to ART drugs (Zhang et al., 2019), where clinical ART resistance is evident. The results for AS from this study were in general agreement with earlier *ex vivo* studies (Quashie et al., 2007; Quashie et al., 2013; Ofori et al., 2021). However, the geometric mean IC_50_ value for DHA was much lower than that from a recent *ex vivo* analysis (Ofori et al., 2021). Although the difference may be due to the use of *in vitro* and *ex vivo* methods, which may not be comparable, the wide ranges of IC_50_ data for both AS and DHA from the *ex vivo* study suggested the presence of parasites with reduced susceptibility to the ART drugs. Furthermore, despite the parasites showing a mean RSA value of <1% indicating susceptibility to ART, there were four parasite isolates showing RSA values slightly higher than 1%, also demanding continuous resistance surveillance, especially in the context of the emergence of ART resistance in East Africa. This study did not detect *pfk13* propeller domain mutations in the 29 parasite isolates. Since most *pfk13* mutations detected earlier in Ghana appeared as low-frequency mutations (Matrevi et al., 2019; Matrevi et al., 2022), molecular surveillance of *pfk13* mutations may require more extensive sampling efforts.

Our *in vitro* drug profiling work showed the overall susceptibility of parasite isolates from Ghana to the ACT partner drugs tested, including LMF, mefloquine, PPQ, naphthoquine, and pyronaridine. For the partner drugs such as mefloquine, LMF, and PPQ, the IC_50_ values were similar to those determined for parasites from other regions in West Africa during the same period (Tinto et al., 2014; Traore et al., 2020). Moreover, these recent clinical isolates showed IC_50_ values within similar ranges of the 3D7 reference strain, and none showed IC_50_ values exceeding cutoff values used to define resistance. Results from the molecular studies further supported the findings from the *in vitro* analysis. The two most widely used ACTs in Ghana, AL and AS-AQ, supposedly exert opposite selection on *pfmdr1* (Okell et al., 2018), with AS-AQ selecting for 86Y and 1246Y (Conrad et al., 2014; Tumwebaze et al., 2015), but AL selecting for the N86 and D1246 wild-type alleles (Sisowath et al., 2005; Humphreys et al., 2007; Zongo et al., 2007; Happi et al., 2009; Some et al., 2010; Baliraine and Rosenthal, 2011; Conrad et al., 2014). The predominant *pfmdr1* 86N/184F/1246D (NFD) haplotype at a prevalence of 69.1% is predicted to confer reduced sensitivity to AL. Nevertheless, our *in vitro* data showed that parasites remained highly sensitive to LMF with IC_50_s ranging from 0.2 to 4.6 nM. It is predicted that simultaneous deployment of multiple first-line ACTs, like the situation in Ghana, may slow down resistance development (Boni et al., 2008).

The *in vitro* drug assay and molecular genotyping results reflected the antimalarial drug policy changes. After the withdrawal of CQ, the *pfcrt* K76T mutation, the major determinant of CQ resistance, and the *pfmdr1* N86Y mutation, which is linked to resistance to CQ and other 4-aminoquinoline drugs such as AQ, have continuously declined in Ghana and other regions of Africa (Duah et al., 2013; Abugri et al., 2018; Okell et al., 2018; Mensah et al., 2020; Tuedom et al., 2021). Consistently, we found that most parasite isolates became CQ-sensitive, whereas only one had IC_50_ higher than the 100 nM resistance threshold (**Figure 1**). Although SP was also discontinued as the frontline treatment of malaria, it has been used for IPTp since 2004, while SP-AQ has been used as SMC in children (Mensah et al., 2020). Our *in vitro* assay confirmed that 83% (24/29) of the isolates were highly resistant to pyrimethamine, with IC_50_ values above 900 nM. Genotyping results of the *pfdhfr* and *pfdhps* genes showed the predominant haplotype as the quadruple mutant IRNGK, consistent with other studies conducted in Ghana (Duah et al., 2013; Abugri et al., 2018; Mensah et al., 2020). Although the quintuple mutant IRNGE responsible for high SP failure rates elsewhere in Africa was not detected in this study, the detection of additional mutations in *pfdhps* at positions 436, 581, and 613 warrants further investigation.

As the rate-limiting enzyme of the folate biosynthesis pathway, *pfgch1* gene amplification has been linked to the *pfdhfr* 164L and *pfdhps* K540E mutations in Thailand (Nair et al., 2008; Sugaram et al., 2020; Turkiewicz et al., 2020). Genomic analysis of the world *P. falciparum* populations indicated that *pfgch1* gene and promoter amplifications were more likely present in parasites with more *pfdhfr/dhps* mutations (Turkiewicz et al., 2020), suggesting that *pfgch1* amplification might compensate for the potential fitness cost associated with the *pfdhfr/dhps* mutations (Heinberg et al., 2013). In our study, all isolates with *pfgch1* amplification carried the *pfdhfr* IRN and *pfdhps* S436A mutations, while one also had three additional *pfdhps* mutations (A437G/A581G/A613S). In some countries such as Malawi and Kenya, there is an indication of increased prevalence of the *pfgch1* promoter amplification after the introduction of SP (Turkiewicz et al., 2020). Consistently, compared to an earlier survey in Ghana that detected double-copy *pfgch1* at a 6.3% prevalence (Osei et al., 2018), our current data showed an increased *pfgch1* gene amplification to 17.3%, albeit the sample number was low. While earlier studies have associated *pfgch1* amplification with greater accumulation of *pfdhfr/dhps* mutations without directly measuring susceptibilities to SP, our finding of increased susceptibilities of isolates with *pfgch1* amplification to *in vitro* pyrimethamine is intriguing. This finding is consistent with confirmation of the detrimental effects of high-level *pfgch1* expression (Heinberg et al., 2013), suggesting a complicated relationship between genetic polymorphisms in the folate synthesis pathway for mediating antifolate resistance.

We have observed positive correlations between drugs of the same chemical groups, such as the ART derivatives, the three aminoalcohols (quinine, LMF, and mefloquine), and two 4-aminoquinolines (naphthoquine and pyronaridine). Intriguingly, we also observed modest correlations between ART drugs (DHA and AS) with LMF and pyronaridine. It is noteworthy that Spearman’s rank-order correlation coefficients for eight comparison pairs exceeded 0.4, suggestive of strong correlations (Tumwebaze et al., 2021). In particular, quinine showed strong correlations (*r* ≥ 0.5) with LMF, mefloquine, and CQ (**Figure 2**). The mutations in drug resistance genes such as *pfmdr1* may underlie these correlations. For example, the *pfmdr1* N86Y and D1246Y mutations found in Africa are linked to increased sensitivity to LMF, mefloquine, and ART (Duraisingh et al., 2000a; Duraisingh et al., 2000b; Reed et al., 2000; Mwai et al., 2009; Tumwebaze et al., 2015). *Pfmdr1* amplification, besides mediating mefloquine resistance, also leads to decreased sensitivity to quinine, LMF, and ART (Price et al., 2004; Sidhu et al., 2006). Thus, the extensive use of multiple antimalarial drugs in Ghana and potential cross-resistance among drug components demand continuous drug resistance monitoring.

One limitation of this study is the small sample size, which may not accurately reflect the drug resistance situation in Ghana. Furthermore, the exact origins of the parasite isolates were unknown, complicating the explanation of the results. Earlier clinical, *ex vivo*, and molecular studies have detected substantial variations in drug efficacy (Abuaku et al., 2012; Abuaku et al., 2019), *ex vivo* drug susceptibility (Quashie et al., 2013; Ofori et al., 2021), and prevalence of resistance markers (Matrevi et al., 2019; Mensah et al., 2020) among the three ecological zones of malaria transmission. Future studies with increased sample size and targeted procurement of parasites from the three ecological zones are needed to present a more accurate picture of drug resistance in Ghana. In addition, most mutations studied in *pfcrt* and *pfmdr1* (mediating resistance to 4-aminoquinoline drugs) and *pfdhfr* (mediating resistance to pyrimethamine) either decreased to very low prevalence or approached fixation, preventing us from performing robust phenotype-genotype association analyses. Nevertheless, establishing continuous cultures of Ghananian parasite isolates provides valuable reference parasite strains for longitudinal monitoring of drug resistance and parasite populations from Ghana and West Africa.

In summary, we established long-term cultures of clinical parasite isolates originating from Ghana. *In vitro* profiling of susceptibility to 11 antimalarials showed that overall the parasite isolates were susceptible to ARTs and ACT partner drugs. A continuously declining prevalence of molecular markers associated with CQ resistance was observed, accompanied by an increased prevalence of mutations suggestive of selection by AL. With the widespread use of ACTs and the emergence of ART resistance in East Africa, drug resistance monitoring efforts need to be reinforced to ensure the effectiveness of the frontline antimalarial drugs.

## Data availability statement

The datasets presented in this study can be found in online repositories. The names of the repository/repositories and accession number(s) can be found below: www.ncbi.nlm.nih.gov; Dhps: OP279202-OP279230. Dhfr: OP279231-OP279259. Crt: MZ572585, MZ572586, MZ572588, MZ572598, MZ572603, MZ572604, MZ572610, MZ572611, MZ572619, MZ572624-MZ572626, MZ572633, MZ572647, MZ572657, MZ572685, MZ572692, MZ572703, MZ572715, MZ572718-MZ572721, MZ572726, MZ572737, OP279260-OP279262. K13: MK877272, MK877273, MK877275, MK877286, MK877291, MK877292, MK877300, MK877301, MK877307, MK877308, MK877319, MK877324-MK877327, MK877333, MK877346, MK877357, MK877374, MK877392, MK877398, MK877412, MK877416-MK877419, MK877424, MK877436, OP297930. Mdr1-1: MZ572380, MZ572381, MZ572383, MZ572394, MZ572399, MZ572400, MZ572408, MZ572409, MZ572415, MZ572416, MZ572427, MZ572432-MZ572435, MZ572441, MZ572451, MZ572462, MZ572480, MZ572500, MZ572506, MZ572517, MZ572521-MZ572524, MZ572528, MZ572540, OP297931. Mdr1-2: MZ577631, MZ577632, MZ577634, MZ577645, MZ577650, MZ577651, MZ577659, MZ577660, MZ577666-MZ577668, MZ577683-MZ577686, MZ577692, MZ577702, MZ577713, MZ577731, MZ577751, MZ577757, MZ577768, MZ577772-MZ577775, MZ577779, MZ577791, OP297932.

## Ethics statement

The studies involving human participants were reviewed and approved by Institutional Review Board, Shanglin People’s Hospital. The patients/participants provided their written informed consent to participate in this study.

## Author contributions

LC and ZY conceived the study. WZ, XXL, QY, MD, XSL, XW, WLZ, HZ, KS, and WYZ performed the experiments. WZ, XXL, QY, YA, LA, BA, ND-Q, and LC analyzed the data. LZ, MP, YQ, and YH collected the parasites. WZ, LC, and ZY wrote the first drafts of the manuscript. All authors critically read and edited the manuscript. All authors read and approved the final manuscript. All authors contributed to the article and approved the submitted version.

## Funding

This study was supported by the National Science Foundation of China (31860604 and U1802286), National Institutes of Health, USA (U19AI089672), major science and technology projects of Yunnan Province (2018ZF0081), and International Science and Technology Cooperation-Yunnan International Science and Technology Cooperation Base (202003AE140004), and The introduction of intellectual projects (202105AP130005 and YNZ201901921). YQ was supported by Guangxi Zhuang Autonomous Region Health Commission of Scientific Research Project (Z20190892). WZ was supported by the Education Department Fund of Yunnan Province (2019J1184).Research Project of Health Commission of Guangxi Zhuang Autonomous Region(ZA20221274).

## Acknowledgments

The authors thank all the patients for volunteering to participate in the study.

## Conflict of interest

The authors declare that the research was conducted in the absence of any commercial or financial relationships that could be construed as a potential conflict of interest.

## Publisher’s note

All claims expressed in this article are solely those of the authors and do not necessarily represent those of their affiliated organizations, or those of the publisher, the editors and the reviewers. Any product that may be evaluated in this article, or claim that may be made by its manufacturer, is not guaranteed or endorsed by the publisher.
