# Supplementary material for: In vitro susceptibility profile of Plasmodium falciparum clinical isolates from Ghana to antimalarial drugs and polymorphisms in resistance markers
Source: Front Cell Infect Microbiol. 2022 Oct 14;12:1015957. doi: 10.3389/fcimb.2022.1015957 (PMC9614232; doi:10.3389/fcimb.2022.1015957)
Supplement: Supplementary file 1 [file DataSheet_1.pdf]

Table S1. Primers used in this study

Gene		primers		Reference
pfkelch13	*kelch13*	FWD	TATAACAAGGCGTAAATATTCGTG	1
		REV	TGTGCATGAAAATAAATATTAAAGAAG	
pfcrt	crt-nest1	FWD	CCGTTAATAATAAATACACGCAG	2
		REV	CGGATGTTACAAAACTATAGTTACC	
	crt-nest2	FWD	TGTGCTCATGTGTTTAAACTT	
		REV	CAAAACTATAGTTACCAATTTTG	
pfmdr1	mdr1(1)-nest1	FWD	TTAAATGTTTACCTGCACAACATAGAAAATT	3
		REV	CTCCACAATAACTTGCAACAGTTCTTA	
	mdr1(1)-nest2	FWD	TGTATGTGCTGTATTATCAGGA	
		REV	CTCTTCTATAATGGACATGGTA	
	mdr1(2)-nest1	FWD	AATTTGATAGAAAAAGCTATTGATTATAA	
		REV	TATTTGGTAATGATTCGATAAATTCATC	
	mdr1(2)-nest2	FWD	GAATTATTGTAAATGCAGCTTTA	
		REV	GCAGCAAACTTACTAACACG	
pfdhfr	dhfr-nest1	FWD	GCGGGATCCATGATGGAACAAGTCTGCGAC	4
		REV	GCGAAGCTTTTAAGCAGCCATATCCATTGAAA	
	dhfr-nest2	FWD	GCGGGATCCATGATGGAACAAGTCTGCGAC	
		REV	GCGAAGCTTACACCTACTCCCGTTCGATC	
pfdhps	dhps-nest1	FWD	GCGGAGCTCATGGAAACTATACAAGAACTAAT	4
		REV	GCGAAGCTTGTTATATAAACATCTGATGGTAT	
	dhps-nest2	FWD	GCGGAGCTCATGATACCCGAATATAAGCATA	
		REV	GCGAAGCTTTTACACTTGGTCTATTTTTGTTA	
pfgch1	*gch1*	FWD	ATGAAACACATAATATGGAAGAAAAA	5
		REV	TCCTTTTCATCTATCACAACAAGG	
	seryl-tRNA	FWD	AAGTAGCAGGTCATCGTGGTT	
		REV	TTCGGCACATTCTTCCATAA	

1 Wang, Z. *et al.* Prevalence of K13-propeller polymorphisms in Plasmodium falciparum from China-Myanmar border in 2007-2012. *Malar J* **14**, 168, doi:10.1186/s12936-015-0672-9 (2015).

2 Zhou, R. M. *et al.* Molecular mutation profile of pfcrt in Plasmodium falciparum isolates imported from Africa in Henan province. *Malar J* **15**, 265, doi:10.1186/s12936-016-1306-6 (2016).

3 Li, J. *et al.* Molecular mutation profile of Pfcrt and Pfmdr1 in Plasmodium falciparum isolates from Bioko Island, Equatorial Guinea. *Infect Genet Evol* **36**, 552-556, doi:10.1016/j.meegid.2015.08.039 (2015).

4 Garg, S. *et al.* Novel point mutations in sulfadoxine resistance genes of Plasmodium falciparum from India. *Acta Trop* **110**, 75-79, doi:10.1016/j.actatropica.2009.01.009 (2009).

5 Osei, M. *et al.* Amplification of GTP-cyclohydrolase 1 gene in Plasmodium falciparum isolates with the quadruple mutant of dihydrofolate reductase and dihydropteroate synthase genes in Ghana. *PLoS One* **13**, e0204871, doi:10.1371/journal.pone.0204871 (2018).

Table S2. The *gch1* gene copy number of Ghananian isolates and the standard laboratory strains Dd2 and 3D7

Sample	*gch1* Copy number
Dd2	2.62
3D7	3.22
16-59	0.55
16-60	3.04
16-62	0.80
16-91	0.47
16-97	2.85
16-99	2.88
16-104	0.63
16-105	0.72
16-113	0.42
16-114	0.63
16-129	0.35
16-136	0.31
16-137	0.62
16-140	0.87
16-148	0.85
16-166	1.01
17-30	0.95
17-53	0.31
17-89	1.07
17-107	3.15
17-108	0.60
17-132	0.72
18-3	0.81
18-11	0.31
18-12	0.97
18-14	0.67
18-15	0.77
18-28	0.99
18-60	3.05

Table S3. Comparison between the IC_{50} values (nM) of parasites with *pfmdr1* Y184 or 184**F** for 11 antimalarial drugs

Drugs	IC_{50} values in nM (Mean±SD)		P values
	Y184 (N=8)	184**F** (N=21)	
Dihydroartemisinin	1.0±0.5	1.0±0.4	0.9305[c]
Artemether	2.5 (1.6-3.4)[a]	1.6 (1.3-2.1)[a]	0.0724[b]
Artesunate	4.3 (2.6-5.6)[a]	4.1 (2.4-4.6)[a]	0.3737[b]
Lumefantrine	2.7±0.7	2.6±1.1	0.8678[c]
Mefloquine	17.9±3.1	17.0±4.8	0.6139[c]
Quinine	49.2 (37.6-55.9)[a]	51.7 (34.3-80.6)[a]	0.7326[b]
Piperaquine	4.3±1.1	4.7±1.2	0.3742[c]
Naphthoquine	7.7 (5.8-9.0)[a]	7.5 (5.5-11.8)[a]	0.8051[b]
Pyronaridine	8.9±2.2	8.1±2.7	0.4724[c]
Chloroquine	14.7 (11.9-25.8)[a]	15.1 (13.5-18.1)[a]	0.7124[b]
Pyrimethamine	9976 (265.5-21269)[a]	7292 (2379-23619)[a]	0.4999[b]
PSA(%)#	1.9 (1.7-2.0)[a]	2.0 (1.6-3.1)[a]	0.4934[b]
RSA(%)#	0.8±0.3	0.8±0.2	0.6060[c]

[a] These data are not normally distributed and shown as median (IQR). Data were compared using Mann-Whitney U test ([b]) or t-test [(c)].
RSA and PSA values are percentages (%).

Table S3. Comparison between the IC_{50} values (nM) of parasites with *pfk13* K189 (wild-type) or 189**T/N** (mutant) for artemisinin derivatives

Drugs	IC_{50} values in nM (Mean±SD)		P values
	K189 (N=6)	189**T/N** (N=23)	
Dihydroartemisinin	0.8±0.5	1.1±0.4	0.2148[c]
RSA(%)	0.7±0.3	0.8±0.2	0.1413[c]
Artemether	2.3 (1.5-3.3)[a]	1.6 (1.4-2.2)[a]	0.2833[b]
Artesunate	3.5 (1.6-5.0)[a]	4.2 (3.2-4.6)[a]	0.5911[b]

[a] These data are not normally distributed and shown as median (IQR). Data were compared using Mann-Whitney U test ([b]) or t-test [(c)].
